# Supplementary material for: Beyond the counter: Pharmacists’ preparedness and response strategies in terrorism-related emergencies in Quetta, Pakistan
Source: PLoS One. 2026 Feb 6;21(2):e0342241. doi: 10.1371/journal.pone.0342241 (PMC12880641; doi:10.1371/journal.pone.0342241)
Supplement: S1 File — (DOCX) [file pone.0342241.s001.docx]

**Supplementary File 1: COREQ checklist**

**Consolidated criteria for reporting qualitative studies (COREQ)**

**Study title: Study title: Beyond the Counter: Pharmacists’ Preparedness and Strategies in Terrorism-Related Emergencies in Quetta, Pakistan**

| **Item No** | | **Guide Questions/Description** | **Remarks** | **Reported on Page #** |  |
| --- | --- | --- | --- | --- | --- |
| **Domain 1: Research team and reflexivity** | | | | |  |
| **Personal Characteristics** | | | | |  |
| 1. Interviewer/ facilitator | | Which author/s conducted the interview or focus group? | FUR, SH, QI | 8-9 |  |
| 2. Credentials | | What were the researcher’s credentials? E.g., PhD, MD | FUR : PhD  SH : PhD  QI: PhD | 8-9 |  |
| 3. Occupation | | What was their occupation at the time of the study? | FUR : Hospital Pharmacist  SH : Academics (Lecturer)  QI: Academics (Lecturer) | 8-9 |  |
| 4. Gender | | Was the researcher male or female? | All interviews were carried out by male researchers | N/A |  |
| 5. Experience and training | | What experience or training did the researcher have? | FUR and SH : Certification in qualitative research methods  QI: A researcher with extensive experience in qualitative studies | N/A |  |
| **Relationship with participants** | | | | |  |
| 6. Relationship established | | Was a relationship established prior to study commencement? | No prior personal relationships with participants were reported. | N/A |  |
| 7. Participant knowledge of the interviewer | | What did the participants know about the researcher? e.g. personal goals, reasons for doing the research? | Participants were informed about the researchers’ academic roles and study objectives. | N/A |  |
| 8. Interviewer characteristics | | What characteristics were reported about the interviewer/facilitator? e.g. Bias, assumptions, reasons and interests in the research topic | The interviewers were pharmacists with prior qualitative research experience | 1 |  |
| **Domain 2: study design** | | | |  |  |
| **Theoretical framework** | | | |  |  |
| 9. Methodological orientation and Theory | What methodological orientation was stated to underpin the study? e.g. grounded theory, discourse analysis, ethnography, phenomenology, content analysis | Phenomenological-based, thematic content analysis. | 9 |  |  |
| **Participant selection** | | | |  |  |
| 10. Sampling | How were participants selected? e.g., purposive, convenience, consecutive, snowball | The participants were purposively selected. | 8 |  |  |
| 11. Method of approach | How were participants approached? e.g., face-to-face, telephone, mail, email | The participants were approached face-to-face. | 7 |  |  |
| 12. Sample size | How many participants were in the study? | 10 participants were approached for the interviews. | 10 |  |  |
| 13. Non-participation Setting | How many people refused to participate or dropped out? Reasons? | We approached 14 participants. Four refused as they were busy with their routine work and prior commitments. | 10 |  |  |
| 14. Setting of data collection | Where was the data collected? e.g., home, clinic, workplace | Data were collected at the pharmacists’ workplace. | 9 |  |  |
| 15. Presence of nonparticipants | Was anyone else present besides the participants and researchers? | Only the interviewers and participant were present, ensuring confidentiality of responses | N/A |  |  |
| 16. Description of sample | What are the important characteristics of the sample? e.g. demographic data, date | The important characteristics are presented in Table 1. | 11 |  |  |
| **Data collection** | | | |  | No |
| 17. Interview guide | Were questions, prompts, and guides provided by the authors? Was it pilot tested? | A semi-structured interview guide was developed, and pilot tested with 3 pharmacists. Data of the pilot phase was not included in the final analysis. | 8 |  |  |
| 18. Repeat interviews | Were repeat interviews carried out? If yes, how many? | No repeat interviews were carried. | N/A |  |  |
| 19. Audio/visual recording | Did the research use audio or visual recording to collect the data? | All interviews were audio recorded. | 9 |  |  |
| 20. Field notes | Were field notes made during and/or after the interview or focus group? | SH and QI prepared the field notes during the interviews that assisted with the transcription. | 9 |  |  |
| 21. Duration | What was the duration of the interviews or focus group? | The duration of the in-depth interviews was approximately 30 min. | 9 |  |  |
| 22. Data saturation | Was data saturation discussed? | Yes | Yes |  |  |
| 23. Transcripts returned | Were transcripts returned to participants for comment and/or correction? | Yes, transcripts were return for confirmation of the precision and accuracy of words, ideas, and jargon used during the script analysis. | 9 |  |  |
| **Domain 3: analysis and findings** | | | |  |  |
| **Data analysis** | | | |  |  |
| 24. Number of data coders | How many data coders coded the data? | Four data coders coded the data. | 9 |  |  |
| 25. Description of the coding tree | Did the authors provide a description of the coding tree? | Interviews were coded line-by-line, and an initial list of nodes was developed. Later, this augmented in developing the framework and transcripts were coded accordingly. New emerging nodes were added to the existing list and were categories as emerging themes. | 10 |  |  |
| 26. Derivation of themes | Were themes identified in advance or derived from the data? | All themes were derived from the data. | N/A |  |  |
| 27. Software | What software, if applicable, was used to manage the data? | NVivo® was used to manage the data. | 9 |  |  |
| 28. Participant checking | Did participants provide feedback on the findings? | No | N/A |  |  |
| **Reporting** | | | |  |  |
| 29. Quotations presented | Were participant quotations presented to illustrate the themes/findings? Was each quotation identified? e.g., participant number | Yes, all quotations were cross matched with the respondent’s demographics. | 12-20 |  |  |
| 30. Data and findings consistent | Was there consistency between the data presented and the findings? | Yes | 12-20 |  |  |
| 31. Clarity of major themes | Were major themes clearly presented in the findings? | Yes | 12-20 |  |  |
| 32. Clarity of minor themes | Is there a description of diverse cases or a discussion of minor themes? | Sub themes were identified and are presented and discussed in the manuscript. | 12-20 |  |  |
