# Supplementary material for: Beyond the counter: Pharmacists’ preparedness and response strategies in terrorism-related emergencies in Quetta, Pakistan
Source: PLoS One. 2026 Feb 6;21(2):e0342241. doi: 10.1371/journal.pone.0342241 (PMC12880641; doi:10.1371/journal.pone.0342241)
Supplement: Attachment — Submitted filename: Comments for PLOS.docx [file pone.0342241.s002.docx]

**Title: Beyond the Counter: Pharmacists’ Preparedness and Response Strategies in Terrorism-Related Emergencies in Quetta, Pakistan**

Dear editor

I would like to express my sincere gratitude for the opportunity to review the manuscript, "Beyond the Counter: Pharmacists’ Preparedness and Response Strategies in Terrorism-Related Emergencies in Quetta, Pakistan." It was an insightful and timely study that tackles an important issue in disaster management. Furthermore, this manuscript addresses a critical issue in healthcare, especially in regions like Pakistan, where terrorism-related emergencies continue to place immense strain on healthcare systems. The role of pharmacists in disaster management is often overlooked, and this study will fill an important gap in the literature.

Thank you for entrusting me with this task. I appreciate the thoughtful process involved in reviewing such a significant piece of research. After a thorough assessment, my detailed comments are provided, and I am proposing “*minor comments*” for the submitted manuscript.

**Title:**

The title accurately reflects the study's focus on pharmacists' preparedness and response to terrorism-related emergencies (TRDs) in Quetta, Pakistan. It is clear, descriptive, and concise. No changes are needed here.

**Abstract:**

The abstract provides a solid overview of the study, outlining the aims, methods, findings, and conclusions. However, the following enhancements can make it clearer and more impactful:

a) The abstract could mention the specific gap in the existing literature that the study addresses. This would help contextualize the research and highlight its novelty.
b) The key findings should be emphasized more directly. Specifically, the lack of formal training in disaster management and the reliance on experiential learning should be presented more prominently.
c) If the word counts allow, briefly mention the key recommendations, particularly those related to curriculum reform, disaster drills, and expanding pharmacists’ roles. This would give the reader a sense of the study’s practical contributions right from the start.

**Introduction:**

The introduction does a good job of presenting the importance of the study. However, the following suggestions could strengthen its clarity and context:

a) The research question could be more clearly articulated. While it is implied, explicitly stating the study's aim to explore pharmacists' preparedness and their response strategies in TRDs could help set the stage more clearly. A possible revision could be: "This study investigates the preparedness, experience, and specific response strategies of pharmacists in managing terrorism-related emergencies at the Trauma Centre of Sandeman Provincial Hospital, Quetta, Pakistan." However, I leave the decision to the authors' judgment.

**Literature review:**

a) The introduction highlights the broader context of terrorism-related disasters (TRDs), but a deeper discussion of the existing research on pharmacists' roles in disaster management could be beneficial. The authors must mention the gap in knowledge regarding the involvement of pharmacists in TRD management, which this study aims to address.
b) It would be beneficial to emphasize why this study is significant, particularly in the context of the increasing frequency of TRDs in Pakistan and the importance of involving pharmacists in disaster response. A stronger argument could be made about the lack of research on pharmacists' roles in disaster management in Pakistan and other conflict-affected regions.

**Methodology:**

The methodology is largely well-structured, but the following improvements can enhance its clarity and rigor:

a) **Qualitative approach justification**

Although the qualitative approach is suitable, a convincing justification for why it is the best method for this study would improve the study. For instance, discuss how qualitative interviews allow for an in-depth understanding of the pharmacists’ experiences and responses during TRDs, which quantitative methods would not capture. For example, "given the complexity of the subject and the limited prior research, a qualitative approach allows for the exploration of nuanced experiences and responses of pharmacists to TRDs."

b) **Sampling and analysis**

Can the authors explain why pharmacists on rotations or daily wages were excluded. Was it the case that individuals with continuous employment would have more consistent exposure to TRDs, making their perspectives more pertinent?

NVivo is used for thematic content analysis, which is a strong choice. However, a deeper discussion on how NVivo helped in organizing, coding, and validating the data would improve the methodology section. Consider explaining the process of data coding and how themes emerged in more detail.

**Results:**

The results section is comprehensive and presents the findings in an organized manner. However, a few enhancements can improve clarity and depth:

a) **Clarity of themes**

While the results are structured around five major themes, it could be helpful to directly tie the findings back to the research questions. For example, each theme can be linked to how it contributes to understanding pharmacists' preparedness and response in TRDs.

While the quotes from pharmacists are powerful, some quotes could be more closely tied to specific themes. For example, linking the quotes to the challenges or barriers they describe could provide a stronger connection between the data and the findings. This would help illustrate how each theme is grounded in the pharmacists' real-world experiences.

**Discussion**

The discussion highlights the key findings and places them in context. I believe that the following changes could strengthen it:

a) Although the authors have provided detailed information, the gaps, particularly in pharmacists' preparedness compared to studies from other countries or regions, should be further explained and especially in areas affected by terrorism. This would help readers better understand how the findings relate to the global context of disaster management."
b) I agree with the recommendations for enhancing disaster management training and the pharmacists' role in response. I would appreciate hearing the authors' perspective on their specific recommendations and how they envision implementing these suggestions within the current healthcare system.

**Recommendations for policy and practice**

The recommendations section is strong but could be made more actionable. Especially, discussion on how to integrate disaster management into pharmacy curricula can be useful.

**Conclusion**

The conclusion effectively summarizes the key findings. Just a suggestion “the findings underscore the need for healthcare systems in conflict zones to expand disaster management roles to include pharmacists more comprehensively, improving both immediate responses and long-term recovery efforts."

**References**

The references are comprehensive and relevant. However, it is important to ensure consistency in formatting (e.g., author names, journal titles, and DOIs).
