# Supplementary material for: Beyond the counter: Pharmacists’ preparedness and response strategies in terrorism-related emergencies in Quetta, Pakistan
Source: PLoS One. 2026 Feb 6;21(2):e0342241. doi: 10.1371/journal.pone.0342241 (PMC12880641; doi:10.1371/journal.pone.0342241)
Supplement: Attachment — Submitted filename: Comments_file (qualitative_study).docx [file pone.0342241.s003.docx]

**Beyond the Counter: Pharmacists’ Preparedness and Response Strategies in Terrorism-Related Emergencies in Quetta, Pakistan**

**Reviewer comments:**

Comment 1:

Line 139. The COREQ statement should be included under the subheading "study design" for clarity.

Comment 2:

Line 155. "Established in 2016, the TC is an advanced and well-equipped institute that offers emergency healthcare services 24/7 with a special focus on TRDs. The TC is the only designated centre in Balochistan province with a dedicated team of healthcare professionals who are experts in treating critical injuries and major trauma cases, including victims of terrorism."

Provide a reference.

Comment 3:

Line 173: "The guide was constructed in the English language."

Line 186: "The second author (FUR; PhD; Hospital Pharmacist) conducted the interviews at a designated space of the TC."

The language of the interviews conducted is not specified. Kindly clarify that interviews can be conducted in English, Urdu, or any other language. If interviews were held in a language other than English, then explain how the responses were translated into English and what procedures were used to validate the accuracy of the translation.

Comment 4:

Line 219. "The saturation was achieved at the eighth interview, but two additional interviews were conducted to ensure the saturation."

Provide a reference on saturation in qualitative research.

And this line on validation in data saturation was placed under the method section rather than the results section.

Comment 5:

Although the study is qualitative, the authors could include simple descriptive or frequency counts of key themes to improve clarity and qualitative depth.

Comment 6:

Line 279. Accordingly, a pharmacist responded that "the moment we receive an emergency alert, medicine trolleys filled with essential medicines, surgical items, and disposables are placed at the ER, OTs, and wards. The chief pharmacist supervises this process to ensure everything is ready before patients come in. We remain on guard until the situation stabilizes, and all victims are taken care of."

Provide the pharmacist's number and gender for this quote to maintain consistency.

Comment 7:

Line 327. "While the American Society of Health-System Pharmacists recommends pharmacists be actively involved in selecting appropriate medications and managing their supply".

Provide a reference to support this statement.
