# Supplementary material for: Beyond the counter: Pharmacists’ preparedness and response strategies in terrorism-related emergencies in Quetta, Pakistan
Source: PLoS One. 2026 Feb 6;21(2):e0342241. doi: 10.1371/journal.pone.0342241 (PMC12880641; doi:10.1371/journal.pone.0342241)
Supplement: Attachment — Submitted filename: Response to Reviewers.docx [file pone.0342241.s004.docx]

**Response to Reviewers’ Comments**

**Manuscript title: Beyond the Counter: Pharmacists’ Preparedness and Response Strategies in Terrorism-Related Emergencies in Quetta, Pakistan**

**GENERAL COMMENTS**

| **Reviewer 1** | | | |
| --- | --- | --- | --- |
| **S. No** | **Reviewer’s Comments** | **Authors’ response** | **Changes in the manuscript** |
| 1. | The manuscript provides rich qualitative insights, but several themes - especially those related to triage and coordination - would benefit from clearer linkage between participants’ quotes and the broader systemic gaps, helping to strengthen the analytical depth. | We thank the reviewer for this insightful comment. In response, we have strengthened the analytical linkage between participants’ narratives and the underlying systemic and structural gaps, particularly within Theme 3 (Preparing for terrorism-related disaster events) and the subtheme on triage, assessment, and coordination.  Specifically, we revised the Results section to more explicitly interpret participants’ quotations in relation to deficiencies in formal disaster governance, role delineation, and interdepartmental coordination. Additionally, we expanded the Discussion to situate these findings within broader institutional and policy-level gaps, emphasizing how pharmacists’ experiential responses reflect the absence of standardized protocols, training frameworks, and integrated command structures. These revisions enhance analytical depth by moving beyond description to clearer explanation of how individual experiences illuminate systemic shortcomings in terrorism-related disaster management. | **Line 255-451** |
|  | Although the Introduction effectively establishes the significance of terrorism-related disasters in Pakistan, it could be strengthened by more explicitly demonstrating how pharmacists' roles differ from or complement other healthcare professionals in these settings. | Thank you for this insightful comment. We have revised the introduction to more explicitly delineate the unique and complementary roles of pharmacists within multidisciplinary healthcare teams during terrorism-related disasters. Specifically, we added a paragraph highlighting how pharmacists’ responsibilities such as emergency medicine procurement and distribution, pharmacotherapy optimization under resource constraints, prevention of medication errors, and supply chain resilience differ from and support the clinical roles of physicians and nurses. | **Line 125 – 140** |
|  | The Methods section is generally strong, but a more detailed justification for the universal sampling approach—and discussion of how non-participation (4/14 pharmacists) may have influenced findings would improve methodological transparency. | We thank the reviewer for this valuable suggestion. We have expanded the Methods section to provide a clearer justification for the use of a universal sampling approach, emphasizing the small, specialized, and context-specific nature of the study population and the methodological appropriateness of including all eligible pharmacists.  Additionally, we have added a discussion addressing non-participation, clarifying the reasons for non-response, confirming the achievement of data saturation, and acknowledging the potential influence of non-participation on the findings. | **Line 185 – 194** |
|  | The Discussion clearly outlines training and curriculum gaps; however, further comparison with models from other countries that have successfully integrated disaster preparedness into pharmacy education could provide more actionable, evidence-based recommendations. | Thank you for this constructive suggestion. We have strengthened the discussion by adding comparative evidence from countries that have implemented and evaluated pharmacy disaster-preparedness education. | **Line 516 – 528** |
| **Reviewer 2** | | | |
| **S. No** | **Reviewer’s Comments** | **Authors’ response** | **Changes in the manuscript** |
| 2. | As I mentioned in my attached comments, such studies relate to the preparedness of pharmacists in response to the needs of war or terrorism-hit regions. A pharmacist is a healthcare provider and should be ready to tackle all emergencies, regardless of what they are. So, I think the study is a kind of awareness and alerting one. It is well designed and presented. The conclusion effectively summarizes the key findings of the study. | We sincerely thank the reviewer for this thoughtful and encouraging assessment of our work. We appreciate the recognition of the study’s broader relevance in highlighting pharmacists’ preparedness across emergency contexts and its role in raising awareness of the need for comprehensive emergency readiness among pharmacists. | **N/A** |
| **Reviewer 3** | | | |
| **S. No** | **Reviewer’s Comments** | **Authors’ response** | **Changes in the manuscript** |
| 3. | The manuscript “Beyond the Counter: Pharmacists Preparedness and Response Strategies in Terrorism-Related Emergencies in Quetta, Pakistan” presents a qualitative study exploring pharmacists experiences and preparedness during terrorism-related emergencies. Overall, the study addresses an important topic. Please see the attached file for detailed reviewer comments. | We sincerely thank the reviewer for the careful evaluation of our manuscript. We have carefully reviewed all detailed comments provided in the attached file and have revised the manuscript accordingly. We believe these changes have strengthened the manuscript and addressed the reviewer’s concerns comprehensively. | **Please refer to the specific comments section (Reviewer 1 in this case)** |

**SPECIFIC COMMENTS**

| **Reviewer 1** | | | |
| --- | --- | --- | --- |
| **S. No** | **Reviewer’s Comments** | **Authors’ response** | **Changes in the manuscript** |
| 1. | The title accurately reflects the study's focus on pharmacists' preparedness and response to terrorism-related emergencies (TRDs) in Quetta, Pakistan. It is clear, descriptive, and concise. No changes are needed here. | Thank you for your positive feedback. We appreciate the reviewer’s assessment that the title accurately reflects the study’s focus on pharmacists’ preparedness and response to terrorism-related emergencies (TRDs) in Quetta, Pakistan. | **N/A** |
| 2. | The abstract provides a solid overview of the study, outlining the aims, methods, findings, and conclusions. However, the following enhancements can make it clearer and more impactful:  a) The abstract could mention the specific gap in the existing literature that the study addresses. This would help contextualize the research and highlight its novelty.  b) The key findings should be emphasized more directly. Specifically, the lack of formal training in disaster management and the reliance on experiential learning should be presented more prominently.  c) If the word counts allow, briefly mention the key recommendations, particularly those related to curriculum reform, disaster drills, and expanding pharmacists’ roles. This would give the reader a sense of the study’s practical contributions right from the start. | Thank you for these constructive and insightful suggestions. We have carefully revised the abstract to address all three points raised. Please refer to the revised version. | **Line 34-59** |
| 3. | Introduction:  The introduction does a good job of presenting the importance of the study. However, the following suggestions could strengthen its clarity and context:  a) The research question could be more clearly articulated. While it is implied, explicitly stating the study’s aim to explore pharmacists’ preparedness and their response strategies in TRDs could help set the stage more clearly. A possible revision could be: “This study investigates the preparedness, experience, and specific response strategies of pharmacists in managing terrorism-related emergencies at the Trauma Centre of Sandeman Provincial Hospital, Quetta, Pakistan.” However, I leave the decision to the authors’ judgment. | We appreciate the positive comments of the reviewer.   a) We have rephrased the research question and incorporated the reviewer’s suggestion accordingly. | **Line 153 - 155** |
| 4. | The introduction highlights the broader context of terrorism-related disasters (TRDs), but a deeper discussion of the existing research on pharmacists' roles in disaster management could be beneficial. The authors must mention the gap in knowledge regarding the involvement of pharmacists in TRD management, which this study aims to address.  b) It would be beneficial to emphasize why this study is significant, particularly in the context of the increasing frequency of TRDs in Pakistan and the importance of involving pharmacists in disaster response. A stronger argument could be made about the lack of research on pharmacists' roles in disaster management in Pakistan and other conflict-affected regions. | We thank the reviewer for this insightful and constructive comment. We fully agree that a clearer articulation of the existing literature, the knowledge gap, and the significance of the study would strengthen the Introduction. These revisions collectively strengthen the theoretical grounding, contextual relevance, and scholarly contribution of the study. We believe the revised Introduction now more clearly justifies the need for this research and aligns closely with the reviewer’s recommendations. Please refer to the revised version for the changes. | **Line 125 - 140** |
| 5. | Methodology:  The methodology is largely well-structured, but the following improvements can enhance its clarity and rigor:  a) Qualitative approach justification  Although the qualitative approach is suitable, a convincing justification for why it is the best method for this study would improve the study. For instance, discuss how qualitative interviews allow for an in-depth understanding of the pharmacists’ experiences and responses during TRDs, which quantitative methods would not capture. For example, "given the complexity of the subject and the limited prior research, a qualitative approach allows for the exploration of nuanced experiences and responses of pharmacists to TRDs."  b) Sampling and analysis  Can the authors explain why pharmacists on rotations or daily wages were excluded. Was it the case that individuals with continuous employment would have more consistent exposure to TRDs, making their perspectives more pertinent?  NVivo is used for thematic content analysis, which is a strong choice. However, a deeper discussion on how NVivo helped in organizing, coding, and validating the data would improve the methodology section. Consider explaining the process of data coding and how themes emerged in more detail. | The comment is appreciated.   a) The suggestion is added to the revised version. b) Thank you for this important query. Pharmacists on rotations or daily wage/contractual arrangements were deliberately excluded to preserve the methodological rigor and credibility of the qualitative findings. The study aimed to capture in-depth, sustained, and context-specific experiences of pharmacists directly involved in terrorism-related disaster management at the Trauma Centre. Furthermore, including such participants could have introduced experiential heterogeneity, potentially diluting the depth of insights related to institutional preparedness, coordination mechanisms, and role continuity during repeated terrorism-related incidents. c) Thank you for this constructive comment. We have now clarified the role of NVivo in the analytic process. | **Line 157 - 239** |
| 6. | Results:  The results section is comprehensive and presents the findings in an organized manner. However, a few enhancements can improve clarity and depth:  a) Clarity of themes  While the results are structured around five major themes, it could be helpful to directly tie the findings back to the research questions. For example, each theme can be linked to how it contributes to understanding pharmacists' preparedness and response in TRDs.  While the quotes from pharmacists are powerful, some quotes could be more closely tied to specific themes. For example, linking the quotes to the challenges or barriers they describe could provide a stronger connection between the data and the findings. This would help illustrate how each theme is grounded in the pharmacists' real-world experiences. | Thank you for this insightful comment.  a & b) We have strengthened the Results section by explicitly linking each major theme to the study’s research questions, clarifying how the findings contribute to understanding pharmacists’ preparedness and response in terrorism-related disasters. | **Please refer to the Results section, as the added statements are extensive and cannot be presented independently here.** |
| 7. | Discussion  The discussion highlights the key findings and places them in context. I believe that the following changes could strengthen it:  a) Although the authors have provided detailed information, the gaps, particularly in pharmacists' preparedness compared to studies from other countries or regions, should be further explained and especially in areas affected by terrorism. This would help readers better understand how the findings relate to the global context of disaster management."  b) I agree with the recommendations for enhancing disaster management training and the pharmacists' role in response. I would appreciate hearing the authors' perspective on their specific recommendations and how they envision implementing these suggestions within the current healthcare system. | a) This was also advised by reviewer 2. We have incorporated the details as required. The details on how curriculum deficiencies are also added. b) It is always cheerful when reviewers agree with the authors. | **Line 495 – 499** |
| 8. | Recommendations for policy and practice  The recommendations section is strong but could be made more actionable. Especially, discussion on how to integrate disaster management into pharmacy curricula can be useful. | We have added the details as it was also advised by reviewer 2. | **Line 516 – 528** |
| 9. | Conclusion  The conclusion effectively summarizes the key findings. Just a suggestion “the findings underscore the need for healthcare systems in conflict zones to expand disaster management roles to include pharmacists more comprehensively, improving both immediate responses and long-term recovery efforts.” | We are thankful for this suggestion. The statement is incorporated in the revised manuscript. | **Line 545 – 551** |
| 10. | References  The references are comprehensive and relevant. However, it is important to ensure consistency in formatting (e.g., author names, journal titles, and DOIs). | The consistency is ensured and all references are corrected as per PLOS One requirements, | **Line 605 - 731** |

| **Reviewer 2** | | | |
| --- | --- | --- | --- |
| **S. No** | **Reviewer’s Comments** | **Authors’ response** |  |
|  | Line 139. The COREQ statement should be included under the subheading "study design" for clarity. | The COREQ statement is moved as per suggestion. | **Line 158 - 160** |
| 1. | Line 155. "Established in 2016, the TC is an advanced and well-equipped institute that offers emergency healthcare services 24/7 with a special focus on TRDs. The TC is the only designated centre in Balochistan province with a dedicated team of healthcare professionals who are experts in treating critical injuries and major trauma cases, including victims of terrorism."  Provide a reference. | Reference is added. | **Line 180** |
| 2. | Line 173: "The guide was constructed in the English language."  Line 186: "The second author (FUR; PhD; Hospital Pharmacist) conducted the interviews at a designated space of the TC."  The language of the interviews conducted is not specified. Kindly clarify that interviews can be conducted in English, Urdu, or any other language. If interviews were held in a language other than English, then explain how the responses were translated into English and what procedures were used to validate the accuracy of the translation. | The interviews were conducted in English. This is added to the manuscript. | **Line 221 - 222** |
| 3. | Line 219. "The saturation was achieved at the eighth interview, but two additional interviews were conducted to ensure the saturation."  Provide a reference on saturation in qualitative research.  And this line on validation in data saturation was placed under the method section rather than the results section. | The reference has been included. Furthermore, saturation is already addressed in the Methods and Results sections of the submitted version. | **Line 255 - 256** |
| 4. | Although the study is qualitative, the authors could include simple descriptive or frequency counts of key themes to improve clarity and qualitative depth. | The details are already mentioned. Please refer to line number and Table 2. | **N/A** |
| 5. | Line 279. Accordingly, a pharmacist responded that "the moment we receive an emergency alert, medicine trolleys filled with essential medicines, surgical items, and disposables are placed at the ER, OTs, and wards. The chief pharmacist supervises this process to ensure everything is ready before patients come in. We remain on guard until the situation stabilizes, and all victims are taken care of."  Provide the pharmacist's number and gender for this quote to maintain consistency. | The pharmacist number and gender is added as suggested. | **Line 313** |
| 6. | Line 327. "While the American Society of Health-System Pharmacists recommends pharmacists be actively involved in selecting appropriate medications and managing their supply".  Provide a reference to support this statement. | The suggested reference has been added. | **Line 355** |
